# Evaluation of rotigotine transdermal patch for the treatment of apathy and motor symptoms in Parkinson’s disease

**DOI:** 10.1186/s12883-016-0610-7

**Published:** 2016-06-07

**Authors:** Robert A Hauser, Jaroslaw Slawek, Paolo Barone, Elisabeth Dohin, Erwin Surmann, Mahnaz Asgharnejad, Lars Bauer

**Affiliations:** Parkinson’s Disease and Movement Disorders Center, USF Health – Byrd Institute, National Parkinson Foundation Center of Excellence, Tampa, FL USA; Department of Neurological–Psychiatric Nursing, Medical University of Gdańsk and Department of Neurology, St Adalbert Hospital, Gdańsk, Poland; Neurodegenerative Disease Center, University of Salerno, Salerno, Italy; UCB Pharma, Brussels, Belgium; UCB Pharma, Monheim am Rhein, Germany; UCB Pharma, Raleigh, NC USA

**Keywords:** Apathy, Parkinson’s disease, Rotigotine transdermal patch, Treatment

## Abstract

**Background:**

This multicenter, double-blind, placebo-controlled study assessed the efficacy of rotigotine transdermal patch on apathy and motor symptoms in patients with Parkinson’s disease (PD).

**Methods:**

Patients with PD-associated apathy (Unified Parkinson's Disease Rating Scale [UPDRS] I item 4 [motivation] ≥2 and patient-rated Apathy Scale [AS] ≥14) were randomized 1:1:1 to “low-dose” rotigotine (≤6 mg/24 h for early PD [those not receiving levodopa] or ≤8 mg/24 h for advanced PD [those receiving levodopa]), “high-dose” rotigotine (≤8 mg/24 h for early PD or ≤16 mg/24 h for advanced PD), or placebo, and maintained at optimal/maximal dose for 12 weeks. Coprimary efficacy variables were: change from baseline to End of Maintenance in patient-rated AS and UPDRS II + III total score. Recruitment was stopped after an interim futility analysis; therefore, all p values are exploratory.

**Results:**

Of 122 patients randomized, 81.1 % completed the study (placebo, n = 32/40 [80.0 %]; low-dose rotigotine, n = 30/41 [73.2 %]; high-dose rotigotine, n = 37/41 [90.2 %]). No treatment difference was observed in the change in patient-rated AS (least squares mean [95 % confidence interval (CI)] difference: low-dose, 0.04 [−2.42, 2.50], p =0.977; high-dose, −0.22 [−2.61, 2.18], p = 0.859). Rotigotine improved UPDRS II + III total scores versus placebo (least squares mean [95 % CI] treatment difference: low-dose, −7.29 [−12.30, −2.28], p = 0.005; high-dose, −6.06 [−10.90, −1.21], p = 0.015), and the “mood/apathy” domain of the Non-Motor Symptom Scale as rated by the investigator (secondary outcome). The most frequent adverse events in rotigotine-treated patients were application site reactions, somnolence, and nausea.

**Conclusions:**

Rotigotine did not improve PD-associated apathy as rated by the patient but provided clinically relevant improvement in motor control and activities of daily living.

**Trial registration:**

ClinicalTrials.gov identifier NCT01782222. Trial registration date: January 30, 2013.

**Electronic supplementary material:**

The online version of this article (doi:10.1186/s12883-016-0610-7) contains supplementary material, which is available to authorized users.

## Background

Apathy is defined as a lack of motivation characterized by diminished goal-oriented behavior and cognition [1]. It is very common in patients with Parkinson’s disease (PD), with prevalence rates ranging from 17 % to 60 % [2–4]. A recent meta-analysis of apathy in PD reported a pooled prevalence of 39.8 % (95 % confidence interval [CI]: 34.6 %, 45.0 %) [3]. Several studies have suggested that apathy is one of the most challenging nonmotor symptoms faced by patients with PD, affecting both quality of life and caregiver burden [3, 5, 6]. Although apathy is observed in both early and advanced stages of PD [7], apathy in PD is associated with older age, depression, cognitive impairment, worse motor symptoms, and more severe disability [3]. Nonetheless, almost half of all apathy in PD occurs in patients without depression or cognitive impairment [3].

While dopamine receptor agonists (DAs) are commonly used to treat the motor symptoms of PD, their effects on the neuropsychiatric symptoms of PD, including apathy, have been less widely studied. In MPTP-lesioned monkeys, dopaminergic dysfunction within the ventral tegmental-nucleus accumbens (VTA-NAcc) pathway has been shown to predict apathetic behaviors, suggesting that DAs might be useful to treat apathy symptoms [8]. Rotigotine is a nonergolinic DA; delivery via a transdermal patch maintains stable plasma levels over 24 hours with a single daily application [9]. In a randomized placebo-controlled study (RECOVER) [10], the Non-Motor Symptom Scale (NMSS) [11, 12] total score, an exploratory outcome, improved with rotigotine compared with placebo, as did individual domain scores for “sleep/fatigue” and “mood/apathy”. A post hoc analysis showed that within the “mood/apathy” domain, there were differences in favor of rotigotine in 4 of 6 validated items: “lost interest in surroundings”, “lost interest in doing things”, “seems sad or depressed”, and “difficulty experiencing pleasure” [13]. Based on these results, we sought to prospectively evaluate rotigotine for the treatment of apathy and motor symptoms in patients with PD and associated apathy.

## Methods

### Overview

PD0005 was a 29-week, multinational, randomized, double-blind, placebo-controlled, 3-arm, phase 4 study that assessed the efficacy of rotigotine on PD-associated apathy and motor symptoms (ClinicalTrials.gov: NCT01782222). Randomization was stratified by disease stage at baseline: early-stage PD (defined as those not taking levodopa) or advanced-stage PD (defined as those taking levodopa). Patients were randomized 1:1:1 to low-dose rotigotine (up to 6 mg/24 h for early PD and 8 mg/24 h for advanced PD, as per dosing recommendations in the United States), high-dose rotigotine (up to 8 mg/24 h for early PD and 16 mg/24 h for advanced PD, as per dosing recommendations elsewhere), or placebo. The study included a screening period of up to 4 weeks, a titration period of up to 4 weeks for early PD and up to 7 weeks for advanced PD, a 12-week maintenance period, a de-escalation period of up to 12 days, and a safety evaluation 28 days after the last dose of study medication. An interim analysis for futility was planned after approximately 120 patients had been randomized and provided data for the coprimary outcome measure (patient-rated Apathy Scale [AS] score change from baseline to End of Maintenance [EoM]).

Patients were enrolled at 19 sites in the United States and 11 sites in Europe: Austria (2 sites), Hungary (2 sites), Poland (3 sites), Slovakia (3 sites), and Spain (1 site) from February 2013 to March 2014.

Patients were assigned to treatment using a computer-generated randomization allocation schedule prepared by UCB Pharma and implemented through an interactive voice/web response system. Investigators, site staff, patients, and monitoring personnel remained blinded to treatment allocation throughout the study.

### Ethics, consent, and permissions

The study was conducted in accordance with principles of Good Clinical Practice and the Declaration of Helsinki, and was approved by appropriate institutional review boards and ethics committees (Additional file 1). Written informed consent was obtained from each patient prior to participation.

### Patients

Key inclusion criteria included: ≥18 years old at screening; diagnosis of PD defined by bradykinesia plus at least 1 of the following: resting tremor, rigidity, or postural impairment, and without any other known or suspected causes of parkinsonism; unsatisfactory control of PD motor symptoms under current treatment; Hoehn-Yahr stage 1–4 in the “ON” state; if taking levodopa, on a stable dose ≥200 mg/day (in combination with benserazide or carbidopa) for at least 28 days prior to baseline; apathy associated with PD present for ≥3 months with Unified Parkinson's Disease Rating Scale (UPDRS) Part I item 4 score (motivation) ≥2 and mean AS score ≥14 as rated by the patient; and Mini Mental State Examination score ≥25.

Key exclusion criteria included: atypical or secondary parkinsonism; history of deep brain stimulation; prior DA therapy within 28 days of baseline; previous discontinuation of a DA (after sufficient duration at adequate dose) owing to lack of efficacy as assessed by the investigator; evidence of an impulse control disorder according to the modified Minnesota Impulsive Disorders Interview (mMIDI); severe depression (Beck Depression Inventory Second Edition [BDI-II] score ≥29); lifetime history of suicide attempt or suicidal ideation in past 6 months; current psychotherapy or behavior therapy; lactating or pregnant; and substance abuse in the past 6 months.

Monoamine oxidase B inhibitors, anticholinergic agents, entacapone, amantadine, and central nervous system therapy (e.g., sedatives, hypnotics, antidepressants, anxiolytics) were permitted if at stable doses for at least 28 days prior to baseline, and expected to be maintained for the duration of the study. Prohibited medications included DAs, dopamine-modulating or -releasing substances, neuroleptics (except clozapine and quetiapine), monoamine oxidase A inhibitors, α-methyldopa, metoclopramide, budipine, and tolcapone.

### Study medication

Rotigotine transdermal patches and matching placebo were supplied by UCB Pharma (Monheim am Rhein, Germany). Active patches released 2 mg/24 h (10 cm^2^), 4 mg/24 h (20 cm^2^), 6 mg/24 h (30 cm^2^), or 8 mg/24 h (40 cm^2^). During the study patients applied 1 to 3 patches per day depending on their assigned daily dose. Rotigotine (or placebo) was administered once daily, starting at 2 mg/24 h in patients with early PD and 4 mg/24 h in patients with advanced PD. Doses were then uptitrated in weekly increments of 2 mg/24 h per week until the optimal or maximal dose was reached. The dose of study medication was considered optimal if both the patient and the investigator felt that PD symptoms, including PD-associated apathy, were adequately controlled. The patient then entered the 12-week maintenance period on the optimal or maximal assigned dose. If during titration an adverse event (AE) occurred that was thought to be due to study medication (excessive dopaminergic stimulation), 1 back-titration to the previous dose level was allowed and the patient was then entered into the maintenance phase on that dose. No dose changes were permitted during the maintenance period. Following the 12-week maintenance period, patients de-escalated their study medication dose by 2 mg/24 h every other day.

### Efficacy assessments

Following baseline evaluations, patients entered the titration period and were contacted by phone at day 7 and evaluated in person on days 14, 21, 28, 35, and 42, as appropriate for the duration of their dose titration. They were then evaluated in person on maintenance days 1, 29, and 85 (EoM). A follow-up safety visit was conducted 28 days following medication withdrawal. Patients who withdrew prematurely were asked to return for a withdrawal visit.

Efficacy assessments performed at baseline and maintenance days 1, 29, and 85 (EoM) included the AS [14] as rated by the patient, AS as rated by the caregiver (if available, and with the same caregiver throughout), UPDRS Parts II + III, Snaith–Hamilton Pleasure Scale (SHAPS), and the 8-item Parkinson's Disease Questionnaire (PDQ-8). Efficacy assessments performed at baseline and maintenance day 85 (EoM) included the NMSS [11, 12], Fatigue Severity Scale (FSS), BDI-II, Montreal Cognitive Assessment, Clinical Global Impression (CGI) item 1, and Patient Global Impression of Change.

The AS [14] is an abbreviated version of the Apathy Evaluation Scale [15] developed specifically for patients with PD, and with proven reliability and validity in assessing apathy in patients with PD. The AS consists of 14 questions that are answered by the patient or caregiver (where appropriate) on a 4-point scale. The total AS score is calculated by summing the single scores, with higher scores indicating more severe apathy; a total score of ≥14 is indicative of clinically relevant apathy symptoms.

### Coprimary, secondary, and other efficacy variables

The coprimary efficacy variables were the change from baseline to EoM in the (1) AS score as rated by the patient and (2) UPDRS II + III total score. Secondary efficacy variables were change from baseline to EoM in the AS score as rated by the caregiver (where available), PDQ-8, SHAPS, and UPDRS III during “ON”, and change from baseline to End of Treatment (EoT; combined data from EoM visit and early withdrawal visit) in the “mood/apathy” domain of the NMSS, BDI-II, and CGI item 1 (severity of illness). Other efficacy variables included change from baseline to EoM in UPDRS II during “OFF”, change from baseline to EoT in NMSS total score, individual NMSS domains excluding “mood/apathy”, FSS, and Montreal Cognitive Assessment. Patient Global Impression of Change at EoT also was evaluated.

### Post hoc analysis of NMSS “mood/apathy” domain

A post hoc analysis was conducted to assess the change from baseline to EoT in the 6 items that comprise the “mood/apathy” domain: item 7 “lost interest in surroundings”, item 8 “lost interest in doing things”, item 9 “feels nervous, worried for no reason”, item 10 “seems sad or depressed”, item 11 “has flat moods”, and item 12 “difficulty experiencing pleasure”. In addition, the results of the 4 items relating specifically to apathy (items 7, 8, 11, and 12) were combined and assessed.

### Safety

Safety was assessed by review of AEs, laboratory values, vital signs, and electrocardiograms. Additional safety assessments included the Columbia-Suicide Severity Rating Scale, which was conducted at every in-person visit, and the mMIDI, which was conducted at maintenance days 1 and 85, and at the follow-up safety visit. Analysis of safety variables was performed on the Safety Set, which included all randomized patients who received at least 1 dose of study medication.

### Statistical analyses

The Full Analysis Set (FAS) was used for analyses of the coprimary, secondary, and other efficacy variables, and included all patients who were randomized, received at least 1 dose of study medication, and had valid primary efficacy baseline measurements and at least 1 valid post-baseline maintenance or valid withdrawal primary efficacy measurement for both primary efficacy variables. Missing data were imputed using last observation carried forward, except for variables that were assessed at only baseline and EoM. The treatment comparison of primary interest was each rotigotine dose group versus placebo. Estimates of treatment effect for the coprimary efficacy variables were obtained from an analysis of covariance (ANCOVA) model that included treatment and disease stage as factors, and baseline value as covariate. For each pairwise comparison of active treatment with placebo, a 2-sided *t* test based on least squares (LS) means with 95 % CI was performed. Secondary variables and the post hoc analysis of the single items of the NMSS “mood/apathy” domain were analyzed according to a similar ANCOVA model as used for the primary variables. As recruitment into the study was stopped after an interim futility analysis, all p-values are exploratory. Other efficacy variables are presented descriptively.

### Power calculations

The study was powered for the coprimary efficacy variables. For the AS, an anticipated clinically meaningful difference between rotigotine and placebo of 3.0 for change from baseline to EoM and a standard deviation (SD) of 8.0 were assumed based on previous studies [16, 17]. A sample size of 151 patients per treatment arm would permit detection of a difference between rotigotine and placebo with 90 % power and a 2-sided α = 0.05, resulting in a sample size of 453 patients in the FAS. For UPDRS II + III total score, an anticipated clinically meaningful difference between rotigotine and placebo of 3.5 for change from baseline to EoM and a SD of 9.6 were assumed [10]. A sample size of 160 patients per treatment group would permit detection of a difference between rotigotine and placebo with 90 % power and a 2-sided α = 0.05, resulting in a sample size of 480 patients in the efficacy population (FAS).

### Interim analysis

When approximately 120 patients were randomized, enrollment was to be stopped and an interim analysis for futility performed by an independent statistician. The interim analysis investigated only the patient-rated AS score change from baseline to EoM. Other efficacy variables, including the other primary efficacy outcome variable (UPDRS II + III total score), were not considered. The conditional power under the current trend (CPtrend) [18] was calculated for each of the treatment arms. The CPtrend refers to the probability of concluding a positive result upon completion of the study, assuming that the population included in the interim analysis is representative of the complete study population.

The continuation criteria for each treatment arm were defined by applying the following conditions to change in AS score from baseline to EoM:

- Treatment difference favoring rotigotine versus placebo ≥2.5 points
- CPtrend ≥60 %.

The decision plan for the interim analysis indicated that if these conditions were met for both the high- and low-dose rotigotine groups, the study would continue unchanged; if these conditions were met for the high-dose but not the low-dose rotigotine group, the study would continue for the high-dose and placebo arms only; and if these conditions were not met for the high-dose rotigotine group, the study would be stopped.

## Results

The interim analysis was performed when the study had randomized 122 patients. The FAS for the interim analysis included 120 patients (40 patients in each treatment group). Continuation criteria for the high-dose rotigotine group were not met and, consequently, the study was stopped. Therefore, all analyses provided should be considered descriptive and all p values considered exploratory.

### Patients

**Fig. 1:**
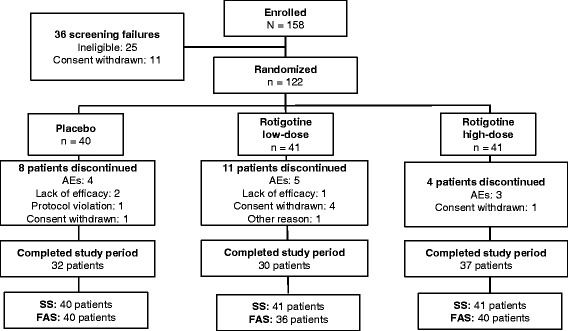
Patient disposition. Abbreviations: AE: adverse event; FAS: Full Analysis Set; SS: Safety Set

A total of 158 patients were enrolled and 122 were randomized to low-dose rotigotine (n = 41), high-dose rotigotine (n = 41), or placebo (n = 40) (Fig. 1). Fifty-six patients were randomized in Europe and 66 in the United States. Ninety-nine patients completed the study.

Baseline demographics and patient characteristics are presented in Table 1. Mean (SD) age was 69.1 (10.1) years and mean time from diagnosis was 4.5 (4.0) years. Twenty-six (21.3 %) patients were early stage (i.e., not receiving levodopa) and 96 (78.7 %) were advanced stage (i.e., receiving levodopa). Mild depression (BDI-II ≥14) was present in 57 (46.7 %) patients. Baseline demographics and patient characteristics were similar across groups, except that time since diagnosis was longer in the rotigotine groups (low-dose = 4.9 years, high-dose = 4.8 years) compared with placebo (3.7 years).

**Table 1:** Demographics and baseline characteristics (Safety Set)

Characteristic	Placebo (n = 40)	Rotigotine low dose (n = 41)	Rotigotine high dose (n = 41)
Male, n (%)	22 (55.0)	27 (65.9)	27 (65.9)
Age, mean ± SD, years	69.0 ± 11.7	68.1 ± 10.5	70.2 ± 8.0
Disease stage, n (%)			
Early (i.e., not taking levodopa)	8 (20.0)	9 (22.0)	9 (22.0)
Advanced (i.e., taking levodopa)	32 (80.0)	32 (78.0)	32 (78.0)
With motor fluctuations	18 (45.0)	17 (41.5)	16 (39.0)
Without motor fluctuations	14 (35.0)	15 (36.6)	16 (39.0)
Time since PD diagnosis, mean ± SD, years	3.7 ± 3.7	4.9 ± 4.0	4.8 ± 4.3
Baseline daily levodopa dose, n (%)			
<600 mg/day	19 (47.5)	18 (43.9)	20 (48.8)
≥600 mg/day	13 (32.5)	14 (34.1)	12 (29.3)
Cardinal signs, n (%)			
Bradykinesia	40 (100)	41 (100)	41 (100)
Rigidity	35 (87.5)	37 (90.2)	40 (97.6)
Resting tremor	28 (70.0)	26 (63.4)	33 (80.5)
Postural instability	25 (62.5)	31 (75.6)	24 (58.5)
Hoehn and Yahr stage, n (%)			
0 (no signs of disease)	0	0	0
1 (unilateral disease)	14 (35.0)	4 (9.8)	5 (12.2)
2 (bilateral disease without impairment of balance)	14 (35.0)	13 (31.7)	14 (34.1)
3 (mild to moderate bilateral disease)	11 (27.5)	20 (48.8)	20 (48.8)
4 (severe disability)	1 (2.5)	4 (9.8)	2 (4.9)
5 (wheelchair bound or bedridden unless aided)	0	0	0
Depression at baseline, n (%)			
No/minimal depression^a^	21 (52.5)	23 (56.1)	21 (51.2)
At least mild depression^b^	19 (47.5)	18 (43.9)	20 (48.8)
UPDRS II + III total score, mean ± SD (0–160)^c^	40.3 ± 19.1	44.4 ± 14.3	39.6 ± 12.4
AS as rated by the patient, mean ± SD (0–42)^c^	19.7 ± 3.8	20.1 ± 4.4	20.2 ± 4.8

^a^Baseline Beck Depression Inventory II 0–13

^b^Baseline Beck Depression Inventory II ≥14

^c^Higher scores indicate worse ratings

AS: Apathy Scale; PD: Parkinson’s disease; SD: standard deviation; UPDRS: Unified Parkinson's Disease Rating Scale

Mean (SD) daily study medication dose during the maintenance period was 7.2 (1.1) mg/24 h in the low-dose rotigotine group, 9.9 (3.8) mg/24 h in the high-dose rotigotine group, and 11.6 (4.5) mg/24 h in the placebo group.

### Efficacy

#### Interim analysis

LS mean (standard error) difference for the high-dose rotigotine group versus placebo for change from baseline to EoM in AS score as rated by patients was 0.08 (1.20) and the CPtrend was 0.77 %, thereby causing the study to be discontinued. There were slight differences in the FAS used for the interim analysis (n = 120) and the final efficacy analysis (n = 116). One patient in the high-dose rotigotine group was not included in the FAS for the interim analysis, but was included in the FAS for the final efficacy analysis because of data cleaning that occurred after the interim analysis was performed. Five additional patients (4 in the low-dose group and 1 in the high-dose group) were mistakenly included in the FAS for the interim analysis (but did not have valid baseline and post-baseline measurements for both primary efficacy variables) and are therefore not included in the FAS for the final efficacy analysis. It was verified that correct assignment of patients to the FAS for the interim analysis would not have modified the decision to stop the study.

### Final analysis

#### Coprimary efficacy variables

**Fig. 2:**
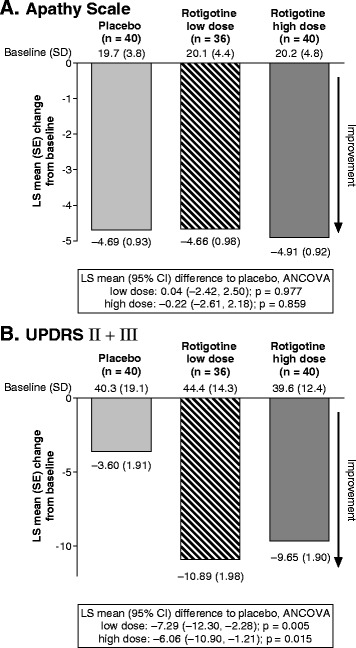
Mean change from baseline to End of Maintenance for coprimary efficacy variables (Full Analysis Set, last observation carried forward): (**a**) Apathy Scale as rated by the patient and (**b**) Unified Parkinson's Disease Rating Scale II + III total score. Abbreviations: ANCOVA: analysis of covariance; CI: confidence interval; LS: least squares; SD: standard deviation; SE, standard error

Results of the ANCOVA for change from baseline to EoM in patient-rated AS are provided in Fig. 2a. Neither low-dose nor high-dose rotigotine was associated with a relevant improvement versus placebo (low-dose LS mean difference to placebo, 0.04, p = 0.977; high-dose LS mean difference to placebo, −0.22, p = 0.859). Results of the ANCOVA for change from baseline to EoM in UPDRS II + III total scores are provided in Fig. 2b. Similar benefits were observed for both low- and high-dose rotigotine versus placebo (low-dose LS mean difference to placebo, −7.29, p = 0.005; high-dose LS mean difference to placebo, −6.06, p = 0.015).

#### Secondary efficacy variables

No marked differences (exploratory p-values >0.05) were observed between rotigotine and placebo for AS as rated by the caregiver, PDQ-8, SHAPS, or BDI-II (Table 2). UPDRS III “ON” scores improved in both low-dose (p = 0.014) and high-dose (p = 0.013) rotigotine groups compared with placebo (Table 2). Overall, CGI severity scores shifted to slightly better categories in all treatment arms.

**Table 2:** Mean change from baseline to End of Maintenance/End of Treatment for secondary efficacy variables (Full Analysis Set)

Assessment (possible score range)^a^	Mean ± SD baseline score			LS mean ± SE change from baseline			LS mean (95 % CI) difference to placebo	
	Placebo (n = 40)	Rotigotine low dose (n = 36)	Rotigotine high dose (n = 40)	Placebo (n = 40)	Rotigotine low dose (n = 36)	Rotigotine high dose (n = 40)	Rotigotine low dose (n = 36)	Rotigotine high dose (n = 40)
Secondary efficacy variables								
AS as rated by caregiver (0–42)^b^	18.4 ± 8.1	19.3 ± 6.2	19.6 ± 6.9	−2.50 ± 1.97	−5.71 ± 2.30	−5.55 ± 2.05	−3.20 (−8.17, 1.76) p = 0.200	−3.04 (−8.19, 2.10) p = 0.239
PDQ-8 total (0–100)^b^	29.1 ± 20.1	27.3 ± 18.3	31.6 ± 18.1	−3.29 ± 2.42	−5.37 ± 2.54	−8.34 ± 2.39	−2.09 (−8.48, 4.31) p = 0.519	−5.06 (−11.29, 1.17) p = 0.111
NMSS “mood/apathy” domain (0–72)^c^	13.5 ± 10.7	15.8 ± 11.2	15.6 ± 11.8	−4.84 ± 1.41	−8.46 ± 1.50	−8.72 ± 1.37	−3.62 (−7.39, 0.15) p = 0.060	−3.88 (−7.46, −0.30) p = 0.034
SHAPS (0–14)^b^	1.6 ± 2.1	2.1 ± 2.4	2.1 ± 2.5	−1.09 ± 0.29	−1.48 ± 0.31	−1.08 ± 0.29	−0.38 (−1.16, 0.40) p = 0.334	0.02 (−0.74, 0.77) p = 0.968
BDI-II total (0–63)^c^	12.8 ± 7.0	13.9 ± 7.2	12.0 ± 5.8	−2.66 ± 0.93	−2.50 ± 1.00	−2.99 ± 0.90	0.16 (−2.34, 2.66) p = 0.899	−0.33 (−2.68, 2.03) p = 0.785
UPDRS Part III in “ON” (0–108)^b^	27.5 ± 14.0	31.6 ± 10.7	27.0 ± 8.4	−3.13 ± 1.50	−8.09 ± 1.55	−7.96 ± 1.49	−4.96 (−8.91, −1.01) p = 0.014	−4.83 (−8.63, −1.03) p = 0.013
Post hoc analysis of single items from NMSS “mood/apathy” domain								
Item 7: Lost interest in surroundings (0–12)	2.2 ± 2.1	2.5 ± 2.5	2.6 ± 3.0	−1.12 ± 0.306	−1.78 ± 0.325	−1.64 ± 0.297	−0.66 (−1.48, 0.16) p = 0.1137	−0.52 (−1.30, 0.25) p = 0.1839
Item 8: Lost interest in doing things (0–12)	3.7 ± 3.1	3.9 ± 2.7	4.4 ± 3.4	−0.96 ± 0.465	−1.97 ± 0.495	−2.41 ± 0.452	−1.01 (−2.25, 0.23) p = 0.1093	−1.45 (−2.63, −0.26) p = 0.0173
Item 11: Has flat moods (0–12)	1.7 ± 1.9	2.7 ± 2.6	2.6 ± 3.2	−1.11 ± 0.283	−1.43 ± 0.301	−1.61 ± 0.274	−0.32 (−1.08, 0.45) p = 0.4110	−0.49 (−1.22, 0.23) p = 0.1785
Item 12: Difficulty experiencing pleasure (0–12)	2.7 ± 3.3	2.6 ± 2.9	2.2 ± 2.2	−0.97 ± 0.342	−1.78 ± 0.365	−1.31 ± 0.332	−0.81 (−1.72, 0.11) p = 0.0823	−0.34 (−1.21, 0.53) p = 0.4411
Combined score of the 4 apathy items: 7, 8, 11, and 12 (0–48)	10.3 ± 7.9	11.7 ± 8.3	11.8 ± 9.5	−3.97 ± 0.979	−7.11 ± 1.043	−7.06 ± 0.952	–3.14 (−5.76, −0.51) p = 0.0196	−3.09 (−5.58, −0.60) p = 0.0156
Item 9: Feels nervous, worried for no reason (0–12)	1.0 ± 1.5	1.7 ± 2.1	1.8 ± 3.0	−0.23 ± 0.324	−0.01 ± 0.345	−0.46 ± 0.315	0.22 (−0.65, 1.09) p = 0.6129	−0.23 (−1.06, 0.60) p = 0.5785
Item 10: Seems sad or depressed (0–12)	2.3 ± 3.3	2.4 ± 3.1	2.1 ± 2.6	−0.75 ± 0.400	−1.23 ± 0.427	−1.14 ± 0.390	−0.48 (−1.55, 0.59) p = 0.3757	−0.38 (−1.40, 0.63) p = 0.4568

^a^Higher scores indicate worse ratings for all assessments

^b^Data are reported as mean change from baseline to End of Maintenance, with last observation carried forward

^c^Data are reported as mean change from Baseline to End of Treatment (combined data from End of Maintenance visit and Early Withdrawal visit), and reported as observed cases

AS: Apathy Scale; BDI-II: Beck Depression Inventory Second Edition; CI: confidence interval; LS: least squares; NMSS: Non-Motor Symptoms Scale; PDQ-8: 8-item Parkinson’s Disease Questionnaire; SHAPS: Snaith–Hamilton Pleasure Scale; SD: standard deviation; SE: standard error; UPDRS: Unified Parkinson’s Disease Rating Scale

The “mood/apathy” domain of the NMSS was improved in the high-dose rotigotine group compared with placebo (p = 0.034), and there was numerical improvement in the low-dose group (Table 2). Post hoc analyses of the 4 apathy items (items 7, 8, 11, 12) of the NMSS demonstrated improvement in the combined score for both the low- and high-dose rotigotine groups compared with placebo (Table 2).

#### Other efficacy variables

The NMSS total score, NMSS “sleep/fatigue” domain, and FSS score showed numerical benefits for rotigotine over placebo (Table 3).

**Table 3:** Mean change from baseline to End of Maintenance/End of Treatment for other efficacy variables (Full Analysis Set)

Assessment (possible score range)^a^	Mean ± SD baseline score			Mean ± SD change from baseline		
	Placebo	Rotigotine low dose	Rotigotine high dose	Placebo	Rotigotine low dose	Rotigotine high dose
	(n = 40)	(n = 36)	(n = 40)	(n = 40)	(n = 36)	(n = 40)
UPDRS Part II in “OFF” (0–52)^b^	12.9 ± 6.3	12.8 ± 5.1	12.7 ± 5.8	−1.5 ± 3.5	−3.5 ± 4.5	−2.6 ± 3.4
NMSS total score (0–360)^c^	50.1 ± 34.1	50.9 ± 31.0	58.2 ± 36.8	−6.7 ± 20.5	−19.5 ± 21.9	−20.7 ± 26.2
NMSS domain score^c^						
Cardiovascular (0–24)	1.7 ± 2.5	1.3 ± 2.4	1.6 ± 2.0	0.3 ± 2.2	0.2 ± 3.1	−0.6 ± 1.6
Sleep/fatigue (0–48)	8.1 ± 5.9	9.9 ± 6.7	10.9 ± 7.5	0.9 ± 7.3	−3.0 ± 7.3	−3.5 ± 6.5
Perception/hallucination (0–36)	0.6 ± 2.0	0.2 ± 1.0	0.4 ± 1.5	0.1 ± 1.6	0.3 ± 1.2	0.1 ± 2.0
Attention/memory (0–36)	5.7 ± 5.2	5.1 ± 4.7	5.6 ± 5.8	−1.2 ± 3.6	−2.2 ± 4.5	0.6 ± 5.9
Gastrointestinal tract (0–36)	3.8 ± 4.1	2.8 ± 3.5	3.9 ± 4.0	0.4 ± 4.0	−0.7 ± 3.8	−1.4 ± 3.6
Urinary (0–36)	6.3 ± 7.6	5.5 ± 6.1	6.7 ± 7.7	−0.6 ± 5.8	−0.3 ± 6.9	−0.7 ± 4.8
Sexual function (0–24)	5.5 ± 7.7	5.9 ± 7.5	6.5 ± 8.7	−1.4 ± 5.8	−2.8 ± 5.4	−2.3 ± 7.6
Miscellaneous (0–48)	5.1 ± 6.5	4.4 ± 6.6	7.0 ± 7.9	−0.7 ± 4.3	−1.2 ± 4.0	−3.2 ± 6.2
FSS (9–63)^c^	41.9 ± 13.09	40.4 ± 11.08	43.6 ± 12.38	−2.8 ± 11.74	−6.6 ± 13.10	−5.7 ± 8.87
MoCA (0–30)^c^	26.4 ± 3.43	25.1 ± 3.29	24.8 ± 4.23	0.2 ± 2.24	0.4 ± 2.58	0.3 ± 3.90
				Score at EoT, n (%)		
PGIC^c^						
Improved	—	—	—	11 (27.5)	14 (38.9)	16 (40.0)
No change	—	—	—	28 (70.0)	16 (44.4)	20 (50.0)
Worsened	—	—	—	0	0	2 (5.0)
Missing	—	—	—	1 (2.5)	6 (16.7)	2 (5.0)

^a^Higher scores indicate worse ratings for all assessments other than MoCA

^b^Data are reported as mean change from Baseline to End of Maintenance, with last observation carried forward

^c^Data are reported as mean change from Baseline to End of Treatment (combined data from End of Maintenance visit and Early Withdrawal visit), and reported as observed cases

FSS, Fatigue Severity Scale; MoCA: Montreal Cognitive Assessment; NMSS: Non-Motor Symptoms Scale; PGIC: Patient Global Impression of Change; SD: standard deviation; UPDRS: Unified Parkinson’s Disease Rating Scale

#### Safety

Most patients completed the study in all treatment groups (placebo, 80.0 %; low-dose rotigotine, 73.2 %; high-dose rotigotine, 90.2 %) (Fig. 1). The most common reason for discontinuation was an AE (placebo, 10.0 %; low-dose rotigotine, 12.2 %; high-dose rotigotine, 7.3 %) (Fig. 1).

Overall incidences of AEs and specific AEs were similar in placebo- and rotigotine-treated patients (Table 4). Most AEs were mild or moderate in severity (Table 4). No deaths were reported. Seven patients experienced serious AEs; 4 of 40 (10.0 %) placebo-treated patients, including abdominal pain, sepsis, cerebrovascular accident, and transient ischemic attack, and 3 of 82 (3.7 %) rotigotine-treated patients, including small intestinal obstruction, ileus, abscess, and cerebral hematoma. Only abdominal pain in the placebo-treated patient was considered to be related to the study drug. There were no clinically relevant mean changes or trends in mean changes for blood pressure, pulse rate, or weight. There were no relevant differences in physical examinations across groups.

**Table 4:** Incidence of TEAEs (Safety Set)^a^

	Placebo	Rotigotine low dose	Rotigotine high dose	Total rotigotine
	(n = 40)	(n = 41)	(n = 41)	(N = 82)
Any TEAE, n (%)	27 (67.5)	28 (68.3)	27 (65.9)	55 (67.1)
Mild	12 (30.0)	16 (39.0)	14 (34.1)	30 (36.6)
Moderate	13 (32.5)	9 (22.0)	12 (29.3)	21 (25.6)
Severe	2 (5.0)	3 (7.3)	1 (2.4)	4 (4.9)
Serious TEAE, n (%)	4 (10.0)	2 (4.9)	1 (2.4)	3 (3.7)
Discontinued due to TEAE, n (%)	4 (10.0)	5 (12.2)	3 (7.3)	8 (9.8)
Drug-related TEAEs, n (%)	18 (45.0)	19 (46.3)	18 (43.9)	37 (45.1)
Deaths, n *(%)*	0	0	0	0
Most common TEAEs ≥5 % in any treatment group^b^				
Application site reactions^c^	3 (7.5)	5 (12.2)	3 (7.3)	8 (9.8)
Nausea	4 (10.0)	4 (9.8)	2 (4.9)	6 (7.3)
Somnolence	3 (7.5)	2 (4.9)	4 (9.8)	6 (7.3)
Depression	2 (5.0)	4 (9.8)	1 (2.4)	5 (6.1)
Fall	2 (5.0)	3 (7.3)	2 (4.9)	5 (6.1)
Constipation	1 (2.5)	2 (4.9)	3 (7.3)	5 (6.1)
Peripheral edema	1 (2.5)	2 (4.9)	3 (7.3)	5 (6.1)
Headache	4 (10.0)	1 (2.4)	3 (7.3)	4 (4.9)
Dyskinesia	2 (5.0)	3 (7.3)	1 (2.4)	4 (4.9)
Fatigue	2 (5.0)	2 (4.9)	1 (2.4)	3 (3.7)
Dry mouth	0	0	3 (7.3)	3 (7.3)
Insomnia	6 (15.0)	1 (2.4)	2 (4.9)	3 (3.7)
Suicidal ideation	3 (7.5)	1 (2.4)	0	1 (1.2)
Rash	2 (5.0)	1 (2.4)	0	1 (1.2)
Tremor	2 (5.0)	1 (2.4)	0	1 (1.2)
Dystonia	2 (5.0)	0	0	0
Vertigo	2 (5.0)	0	0	0
Visual hallucinations	2 (5.0)	0	0	0

^a^Data are number of patients reporting at least 1 adverse event (%)

^b^MedDRA (Version 16) Preferred Term except for application site reactions

^c^Refers to High Level Term “application and instillation site reactions”

TEAE: treatment-emergent adverse event

One of 40 (2.5 %) placebo-treated patients and 3 of 82 (3.7 %) rotigotine-treated patients had a positive finding on 1 mMIDI module, but associated structured interviews were negative in the rotigotine-treated patients and an interview was not conducted in the placebo-treated patient. Some degree of suicidal ideation, as reported on the Columbia-Suicide Severity Rating Scale, was identified in 4 of 40 (10.0 %) placebo-treated patients (1 reported at baseline and 3 during the study) and 5 of 82 (6.1 %) rotigotine-treated patients (all in low-dose rotigotine group; 4 at baseline, 1 during the study).

## Discussion

This was the first placebo-controlled study to prospectively assess the effects of a DA on apathy as a primary outcome measure in patients with PD. The study was discontinued after a preplanned interim analysis because continuation criteria were not met regarding improvement in the AS (as rated by the patient) in rotigotine versus placebo groups. In the final analysis, neither low-dose nor high-dose rotigotine was associated with an improvement in AS scores versus placebo. However, improvement was observed in UPDRS II + III total scores in both rotigotine groups compared with placebo, consistent with rotigotine’s known benefit on motor symptoms and activities of daily living in patients with PD [19–21].

The AS [14] was chosen as the coprimary outcome measure to assess apathy following review of the literature and was in accordance with a recent recommendation by the Movement Disorder Society task force [22]. However, the sensitivity to change of the AS is not known, and PD patients with apathy may have little or no insight into change in their apathy status. For the change in AS as completed by the caregiver, there was a larger numerical difference, with results similar to the anticipated −3.0-point treatment difference that was initially expected for the primary variable, suggesting that caregivers might identify changes of which patients with apathy are unaware.

The “mood/apathy” domain of the NMSS consists of 4 apathy items, 1 mood item, and 1 anxiety item. Notably, in our study, improvement was observed in the combined score of the 4 apathy items from the NMSS, and these items were assessed by the study investigator. This supports the hypothesis that changes in apathy may be more apparent to an observer than to the patient, but it is also possible that these items could be more sensitive to change even if completed by the patient, and this is an important area for future study. The “mood/apathy” domain of the NMSS was improved in the rotigotine RECOVER study [10], as discussed above, and was the primary impetus for this trial. Additionally, in a recent study of rotigotine in PD patients with nonmotor symptoms, although rotigotine did not improve the NMSS total score compared with placebo, the “mood/apathy” domain was improved [23]. Thus, in each of 3 separate studies, one of patients with unsatisfactory early-morning motor symptom control [10], one of patients presenting with nonmotor features [23], and one of patients with apathy and unsatisfactory motor control (current PD0005 study), the “mood/apathy” domain of the NMSS was improved with rotigotine compared with placebo. However, unlike the current study, the sum of the 4 apathy items from the NMSS was not evaluated separately from the full “apathy/mood” domain in the other studies. We also note that the “mood/apathy” domain of the NMSS was an exploratory outcome in all of these studies, and benefit has not been demonstrated prospectively using this measure as a primary outcome. Our experience suggests that the combined 4 apathy items of the NMSS deserves further evaluation as a potential outcome measure for clinical trials of apathy.

PD studies using other assessment scales that also include apathy/motivational items include: a meta-analysis of studies of the nonergolinic DA pramipexole, which suggests that pramipexole may improve mood and motivational items of the UPDRS Part I [24]; and a comparative cross-sectional study, in which the use of pramipexole was associated with lower apathy scores than levodopa or ropinirole, when assessed by the Neuropsychiatric Inventory apathy subscore [25]. Thus, the benefits observed on apathy and motivational items may relate to the specific pharmacological profiles of DA agonists.

In our study, rotigotine was well tolerated, and the safety profile was consistent with the known safety profile of rotigotine. In addition, the overall completion rate was acceptable, indicating that clinical trials evaluating interventions in PD patients with apathy are feasible. However, further investigation is required to delineate the best and most appropriate outcome measures. Apathy remains a burdensome nonmotor feature of PD for which effective treatments are needed.

## Conclusions

In summary, rotigotine did not improve PD-associated apathy as rated by the patient but provided clinically meaningful improvements in motor control and activities of daily living versus placebo. Rotigotine was well tolerated, with no new safety concerns. Further investigation is required to determine the most appropriate outcome measures for clinical trials of apathy in patients with PD.

## Abbreviations

AE, adverse event; ANCOVA, analysis of covariance; AS, Apathy Scale; BDI-II, Beck Depression Inventory Second Edition; CGI, clinical global impression; CI, confidence interval; CPtrend, conditional power under the current trend; DA, dopamine receptor agonist; EoM, End of Maintenance; EoT, End of Treatment; FAS, full analysis set; FSS, Fatigue Severity Scale; LS, least squares; mMIDI, modified Minnesota Impulsive Disorders Interview; NMSS, Non-Motor Symptom Scale; PD, Parkinson’s disease; PDQ-8, 8-item Parkinson’s Disease Questionnaire; SD, standard deviation; SHAPS, Snaith–Hamilton Pleasure Scale; UPDRS, Unified Parkinson’s Disease Rating Scale

## Additional file

Additional file 1: Institutional Review Boards or Independent Ethics Committees of participating sites in the PD0005 study. (PDF 220 kb)
