# Supplementary material for: Evaluation of rotigotine transdermal patch for the treatment of apathy and motor symptoms in Parkinson’s disease
Source: BMC Neurol. 2016 Jun 7;16:90. doi: 10.1186/s12883-016-0610-7 (PMC4895976; doi:10.1186/s12883-016-0610-7)
Supplement: Additional file 1: — Institutional Review Boards or Independent Ethics Committees of participating sites in the PD0005 study. (PDF 220 kb) [file 12883_2016_610_MOESM1_ESM.pdf]

| Site No | Hospital/Institutional Address | IRB/IEC Name and Address |
|---|---|---|
| 3001 | Medizinische Universität Innsbruck<br>Department für Neurologie<br>Anichstrasse 35<br>6020 Innsbruck<br>AUSTRIA | Central IEC<br>Ethikkommission der Medizinischen Universität Innsbruck<br>Geschäftsstelle<br>Innrain 43<br>6020 Innsbruck<br>AUSTRIA |
| 3003 | Landesnervenklinik Wagner Jauregg<br>Neurologische Abteilung<br>Wagner Jauregg Weg 15<br>4020 Linz<br>AUSTRIA | Local IEC<br>Ethikkommission des Landes Oberösterreich<br>Wagner Jauregg Weg 15<br>4020 Linz<br>AUSTRIA<br><br>Central IEC<br>Ethikkommission der Medizinischen Universität Innsbruck<br>Geschäftsstelle<br>Innrain 43<br>6020 Innsbruck<br>AUSTRIA |
| 3301 | Semmelweis Egyetem Általános Orvostudományi Kar<br>Neurológiai Klinika<br>Balassa János utca 6<br>H-1083 Budapest<br>HUNGARY | Central IEC<br>Egészségügyi Tudományos Tanács Klinikai Farmakológiai Etikai Bizottsága<br>Arany János u. 6-8<br>H-1051 Budapest<br>HUNGARY |
| 3302 | Kenézy Kórház és Rendelőintézet<br>Egészségügyi Szolgáltató Kft.<br>Neurológiai Osztály<br>Bartók Béla út 2-26<br>H-4043 Debrecen<br>HUNGARY | Central IEC<br>Egészségügyi Tudományos Tanács Klinikai Farmakológiai Etikai Bizottsága<br>Arany János u. 6-8<br>H-1051 Budapest<br>HUNGARY |
| 3504 | Centrum Medyczne HCP Lecznictwo Stacjonarne<br>Oddzial Udarowy<br>ulica 28 czerwca 1956 r. 194<br>61-485 Poznań<br>Wielkopolskie<br>POLAND | Central IEC<br>Komisja Bioetyczna Przy Okregowej Izbie Lekarskiej w Gdańsku<br>Ulica Śniadeckich 33<br>80-204 Gdańsk<br>POLAND |
| 3506 | Centrum Terapii Innowacyjnych S.C.<br>Ulica Chrzanowskiego 3/5<br>81-338 Gdynia<br>POLAND | Central IEC<br>Komisja Bioetyczna Przy Okregowej Izbie Lekarskiej w Gdańsku<br>Ulica Śniadeckich 33<br>80-204 Gdańsk<br>POLAND |
| 3509 | Centrum Badan Klinicznych PI-House Sp. z o.o.<br>ulica Na Zaspe 3<br>80-546 Gdańsk<br>Pomorskie<br>POLAND | Central IEC<br>Komisja Bioetyczna Przy Okregowej Izbie Lekarskiej w Gdańsku<br>Ulica Śniadeckich 33<br>80-204 Gdańsk<br>POLAND |
| 3801 | Neurologická ambulancia, s. r. o.<br>Nová 21<br>97404 Banská Bystrica<br>SLOVAKIA | Central IEC<br>Nezávislá etická komisia Banskobystrického samosprávneho kraja<br>Úrad Banskobystrického samosprávneho kraja<br>Námestie SNP 23<br>974 01 Banská Bystrica<br>SLOVAKIA |

| | | |
|---|---|---|
| 3806 | Neurologická Ambulancia<br>SNP 25<br>05342 Krompachy<br>SLOVAKIA | Local IEC<br>Etická komisia Košického samosprávneho kraja<br>Námestie Maratónu mieru 1<br>042 66 Košice<br>SLOVAKIA<br><br>Central IEC<br>Nezávislá etická komisia Banskobystrického samosprávneho kraja<br>Úrad Banskobystrického samosprávneho kraja<br>Námestie SNP 23<br>974 01 Banská Bystrica<br>SLOVAKIA |
| 3807 | NEURON - D.T., s.r.o.<br>Hálkova 3<br>010 01Žilina<br>SLOVAKIA | Local IEC<br>Etická komisia Žilinského samopsrávneho kraja<br>Komenského 48<br>011 09 Žilina<br>SLOVAKIA<br><br>Central IEC<br>Nezávislá etická komisia Banskobystrického samosprávneho kraja<br>Úrad Banskobystrického samosprávneho kraja<br>Námestie SNP 23<br>974 01 Banská Bystrica<br>SLOVAKIA |
| 4001 | Hospital de la Santa Creu i Sant Pau<br>C/ Mas Casanovas, 90<br>08041 Barcelona<br>SPAIN | Central IEC<br>Comité Ético de Investigación Clínica de la Fundació de Gestió Sanitària del Hospital de la Santa Creu i Sant Pau<br>Avda. Sant Antoni Mª Claret 167<br>1ª Planta – Izquierda S. Farmacología Clínica<br>08025 Barcelona<br>SPAIN |
| 4302 | University of Nevada School of Medicine<br>1707 West Charleston Blvd.<br>Suite 220<br>Las Vegas, NV 89102<br>UNITED STATES | Local IRB<br>Western Institutional Review Board<br>1019 39th Avenue SE<br>Suite 120<br>Puyallup, WA 98374-2115<br>UNITED STATES |
| 4303 | Parkinson Disease/Movement Disorders<br>283 Commack Road<br>Suite 101<br>Commack, NY 11725<br>UNITED STATES | Central IRB<br>Quorum Review IRB<br>1501 Fourth Avenue<br>Suite 800<br>Seattle, WA 98101<br>UNITED STATES |
| 4306 | Neurology Associates of Ormond Beach<br>8 Mirror Lake Drive<br>Suite A & B<br>Ormond Beach, FL 32174<br>UNITED STATES | Central IRB<br>Quorum Review IRB<br>1501 Fourth Avenue<br>Suite 800<br>Seattle, WA 98101<br>UNITED STATES |
| 4307 | Imaging and Neurosciences Center (INC)<br>University of Utah Dept. of Neurology<br>729 Arapeen Dr.<br>Salt Lake City, UT 84108<br>UNITED STATES | Local IRB<br>University of Utah Institutional Review Board<br>75 South 2000 East<br>Salt Lake City, UT 84112<br>UNITED STATES |

| | | |
|---|---|---|
| 4309 | Pacific Neuroscience Medical Group<br>1701 Solar Drive<br>Suite 140<br>Oxnard, CA 93030<br>UNITED STATES | Central IRB<br>Quorum Review IRB<br>1501 Fourth Avenue<br>Suite 800<br>Seattle, WA 98101<br>UNITED STATES |
| 4311 | Palm Beach Neurological Center,<br>Advanced Research Consultants, Inc.<br>3365 Burns Road<br>Suite 203<br>Palm Beach Gardens, FL 33410<br>UNITED STATES | Central IRB<br>Quorum Review IRB<br>1501 Fourth Avenue<br>Suite 800<br>Seattle, WA 98101<br>UNITED STATES |
| 4312 | University of Cincinnati Physicians Company, LLC.<br>260 Stetson Street<br>Suite 2300 PO Box 670525<br>Cincinnati, OH 45267-0525<br>UNITED STATES | Local IRB<br>University of Cincinnati Institutional Review Board<br>51 Goodman Drive, University Hall<br>Suite 300<br>Cincinnati, OH 45221-0566<br>UNITED STATES |
| 4313 | University of South Florida<br>Parkinson's Disease and Movement Disorders Center<br>4001 E. Fletcher Avenue<br>6th Floor<br>Tampa, FL 33613<br>UNITED STATES | Local IRB<br>Western Institutional Review Board<br>1019 39th Avenue SE<br>Suite 120<br>Puyallup, WA 98374-2115<br>UNITED STATES |
| 4314 | NeuroStudies.net, LLC<br>2665 N. Decatur Road<br>Suite 740 & 440<br>Decatur, GA 30033<br>UNITED STATES | Central IRB<br>Quorum Review IRB<br>1501 Fourth Avenue<br>Suite 800<br>Seattle, WA 98101<br>UNITED STATES |
| 4317 | Mount Sinai School of Medicine<br>5 East 98th Street<br>New York, NY 10029-6574<br>UNITED STATES | Local IRB<br>Biomedical Research Alliance of New York, LLC<br>Institutional Review Board<br>1981 Marcus Avenue<br>Suite 210<br>Lake Success, NY 11042<br>UNITED STATES |
| 4319 | Asheville Neurology Specialists, PA<br>31 Dogwood Road<br>Asheville, NC 28806<br>UNITED STATES | Central IRB<br>Quorum Review IRB<br>1501 Fourth Avenue<br>Suite 800<br>Seattle, WA 98101<br>UNITED STATES |
| 4322 | Iowa Physicians Clinic Medical Foundation<br>1221 Pleasant Street<br>Suite 300<br>Des Moines, IA 50309<br>UNITED STATES | Central IRB<br>Quorum Review IRB<br>1501 Fourth Avenue<br>Suite 800<br>Seattle, WA 98101<br>UNITED STATES |

| | | |
|---|---|---|
| 4324 | Coastal Neurology, PA<br>1833 North Paris Avenue<br>Port Royal, SC 29935<br>UNITED STATES | Central IRB<br>Quorum Review IRB<br>1501 Fourth Avenue<br>Suite 800<br>Seattle, WA 98101<br>UNITED STATES |
| 4325 | The Neurological Institute, PA<br>2607 E. 7th Street<br>Suite 200<br>Charlotte, NC 28204<br>UNITED STATES | Central IRB<br>Quorum Review IRB<br>1501 Fourth Avenue<br>Suite 800<br>Seattle, WA 98101<br>UNITED STATES |
| 4326 | Springfield Neurology Associates<br>300 Carew St.<br>Suite 2<br>Springfield, MA 01104<br>UNITED STATES | Central IRB<br>Quorum Review IRB<br>1501 Fourth Avenue<br>Suite 800<br>Seattle, WA 98101<br>UNITED STATES |
| 4329 | Banner Sun Health Research Institute<br>10515 W. Santa Fe Drive<br>Sun City, AZ 85351<br>UNITED STATES | Local IRB<br>Western Institutional Review Board<br>1019 39th Avenue SE<br>Suite 120<br>Puyallup, WA 98374-2115<br>UNITED STATES |
| 4330 | The Neuroscience Center<br>3603 Bienville Blvd.<br>Suite 102<br>Ocean Springs, MS 39564<br>UNITED STATES | Central IRB<br>Quorum Review IRB<br>1501 Fourth Avenue<br>Suite 800<br>Seattle, WA 98101<br>UNITED STATES |
| 4332 | Medical University of South Carolina,<br>Charleston Memorial Hospital<br>Movement Disorders Program<br>326 Calhoun Street<br>Suite 308<br>McClennan Banks Building<br>Charleston, SC 29401<br>UNITED STATES | Local IRB<br>Medical University of South Carolina/Office of Research Integrity<br>Harborview Tower<br>19 Hagood Avenue<br>Suite 601<br>Charleston, SC 29425<br>UNITED STATES |
| 4335 | Neurological Associates<br>7301 Forest Avenue<br>Suite 300<br>Richmond, VA 23226<br>UNITED STATES | Central IRB<br>Quorum Review IRB<br>1501 Fourth Avenue<br>Suite 800<br>Seattle, WA 98101<br>UNITED STATES |

IRB; Institutional Review Board; IEC, Independent Ethics Committee
